# Solid-state NMR assignment of α-synuclein polymorph prepared from helical intermediate

**DOI:** 10.1007/s12104-024-10188-0

**Published:** 2024-07-04

**Authors:** Sahil Ahlawat, Surabhi Mehra, Chandrakala M. Gowda, Samir K Maji, Vipin Agarwal

**Affiliations:** 1 Tata Institute of Fundamental Research, Sy. No. 36/P, Gopanpally Village, Serilingampally Mandal, Ranga Reddy District, Hyderabad, 500 046 India; 2 Department of Biosciences and Bioengineering, Indian Institute of Technology Bombay, Powai, Mumbai, 400 076 India

**Keywords:** α–Synuclein, Fibrils, Oligomers, Solid-state NMR, Assignment

## Abstract

Synucleinopathies are neurodegenerative diseases characterized by the accumulation of α-synuclein protein aggregates in the neurons and glial cells. Both ex vivo and in vitro α-synuclein fibrils tend to show polymorphism. Polymorphism results in structure variations among fibrils originating from a single polypeptide/protein. The polymorphs usually have different biophysical, biochemical and pathogenic properties. The various pathologies of a single disease might be associated with distinct polymorphs. Similarly, in the case of different synucleinopathies, each condition might be associated with a different polymorph. Fibril formation is a nucleation-dependent process involving the formation of transient and heterogeneous intermediates from monomers. Polymorphs are believed to arise from heterogeneous oligomer populations because of distinct selection mechanisms in different conditions. To test this hypothesis, we isolated and incubated different intermediates during in vitro fibrillization of α-synuclein to form different polymorphs. Here, we report ^13^C and ^15^N chemical shifts and the secondary structure of fibrils prepared from the helical intermediate using solid-state nuclear magnetic spectroscopy.

**Supplementary Information:**

The online version contains supplementary material available at 10.1007/s12104-024-10188-0.

## Biological context

α-Synuclein is an intrinsically disordered protein (IDP) that tends to form aggregates. The accumulation of these aggregates results in neurodegenerative diseases known as synucleinopathies (Martí et al. 2003). Synucleinopathies include Parkinson’s disease (PD), Dementia with Lewy bodies (DLB), multiple systems atrophy (MSA) and pure autonomic failure (PAF) (Spillantini and Goedert 2018). All these diseases have very different symptoms and pathologies despite having a common cause, i.e., deposition of α-synuclein aggregates (Martí et al. 2003; Spillantini and Goedert 2018). In these diseases, α-synuclein accumulates in distinct parts of the human brain. PD and DLB have deposition of aggregates in Lewy bodies (Spillantini et al. 1997) while in the case of MSA, they are deposited in the cytosol of oligodendrocytes (Spillantini et al. 1998). The patients with the same disease also have distinct behaviors and pathologies. These different behaviors of a single disease may also be attributed to polymorphism. Polymorphism is a phenomenon resulting in structural variations in fibrils originating from a single polypeptide/protein. Polymorphs differ in morphological (length, diameter, twists and twist length), biophysical (diffraction pattern and ThT kinetics), biochemical (protease resistance and HDX exchange) and pathogenic (seeding and toxicity) properties (Gallardo et al. 2020). Distinct polymorphs can arise because of variations in protofilaments’ number, relative arrangement and structural differences in monomers. The distinct behavior of synucleinopathies can be due to the formation of a different polymorph in each case.

Polymorphism has been reported for in vitro generated fibrils of α-synuclein, Aβ, tau and other aggregation-prone proteins (Gallardo et al. 2020; Willbold et al. 2021). During in vitro fibril formation, the polymorph observed may depend on buffers, salt concentration, pH, temperature, agitation method, lipids and mechanical handling (Willbold et al. 2021). Both in vitro fibril preparations and fibrils extracted from patients’ brains (ex vivo) show polymorphism (Gath et al. 2011, 2014a; Comellas et al. 2011; Bousset et al. 2013; Verasdonck et al. 2016; Mehra et al. 2022; Schweighauser et al. 2020). Solid-state nuclear magnetic resonance (ssNMR) spectroscopy and cryo-electron microscopy (Cryo-EM) have been employed to determine fibril structures. The structures of in vitro generated fibrils from wild-type, mutant and modified α-synuclein, ex vivo fibril samples from different diseases, fibrils amplified from ex vivo samples and in vitro preparations in the presence of membrane, ions and other proteins (Guerrero-Ferreira et al. 2018, 2019; Li et al. 2018a, b; Ni et al. 2019; Boyer et al. 2019, 2020; Sun et al. 2020, 2021, 2023; Zhao et al. 2020a, b, 2023; Schweighauser et al. 2020; Lövestam et al. 2021; Long et al. 2021; McGlinchey et al. 2021; Tao et al. 2022; Yang et al. 2022, 2023; Frieg et al. 2022; Fan et al. 2023; Zhang et al. 2023; Hu et al. 2024; Dhavale et al. 2024; Chen et al. 2024; Balana et al. 2024). The in vitro generated polymorphs, when injected into the rat brain, resulted in different pathology (Peelaerts et al. 2015). The ex vivo fibrils from distinct diseases have distinct properties and structures (Peng et al. 2018; Shahnawaz et al. 2020; Schweighauser et al. 2020; Yang et al. 2022). The fibrils from different cases of a single disease (MSA) also show minor structural variations (Schweighauser et al. 2020).

Fibril formation is a nucleation-dependent pathway in which monomers combine to form oligomers and these oligomers mature to form fibrils (Radford and Weissman 2012; Breydo and Uversky 2014). The oligomers are transient and heterogeneous species formed during the lag phase of fibril formation (Cremades et al. 2017). Each oligomer species can evolve into a distinct polymorph but not all oligomer species are competent to mature into fibrils. The fibril forming conformations with autocatalytic growth competent activity will populate while all the other oligomer species will get outnumbered (Willbold et al. 2021). This selection process limits in vitro fibril formation to a single polymorph when a single autocatalytic growth competent conformation is dominant. However, if more than one conformations are autocatalytic growth competent, distinct polymorphs can be obtained. It is unclear whether a single autocatalytic growth competent oligomer can result in polymorphs, particularly in cases where distinct polymorphs have similar monomer/filament structure. The secondary nucleation and shearing might also be contributing factors in deciding which polymorph will be populated. Similarly, the selection mechanisms limit the polymorphs during in vivo fibril formation (Willbold et al. 2021).

In our recent investigation, we found that oligomeric species formed during α-synuclein fibril formation demonstrated the capability to evolve into distinct polymorphs under identical conditions (Mehra et al. 2022). We isolated these on-pathway intermediate species, allowed them to mature into fibrils and comprehensively characterized the resulting fibrils using a combination of biophysical techniques and ssNMR methods (Mehra et al. 2022). This manuscript reports the chemical shift assignment of one such α-synuclein polymorph prepared by isolating a helical intermediate and maturing it into fibrils.

## Materials and methods

### Protein expression and purification

U-[^15^N, ^13^C] α-synuclein protein was expressed in *E. coli* BL21 (DE3) strain with pRK172 plasmid in M9 medium containing 2 g/L ^13^C-glucose and 1 g/L ^15^NH_4_Cl as the sole source of carbon and nitrogen, respectively. The protein was purified and lyophilized by the protocol described by Volles et al. (Volles and Lansbury 2007) with minor modifications (Singh et al. 2013). The lyophilized α-synuclein was dissolved in 20 mM glycine-NaOH buffer (pH 7.4, 0.01% NaN_3_). The solution was centrifuged at 14,000 g for 30 min to remove higher-order aggregates. The supernatant was dialyzed against the same buffer overnight at 4˚C. The protein solution was passed through 100 kDa MW cut-off filters and flow-through was taken and used as low molecular weight (LMW).

## Fibril formation

**Fig. 1:**
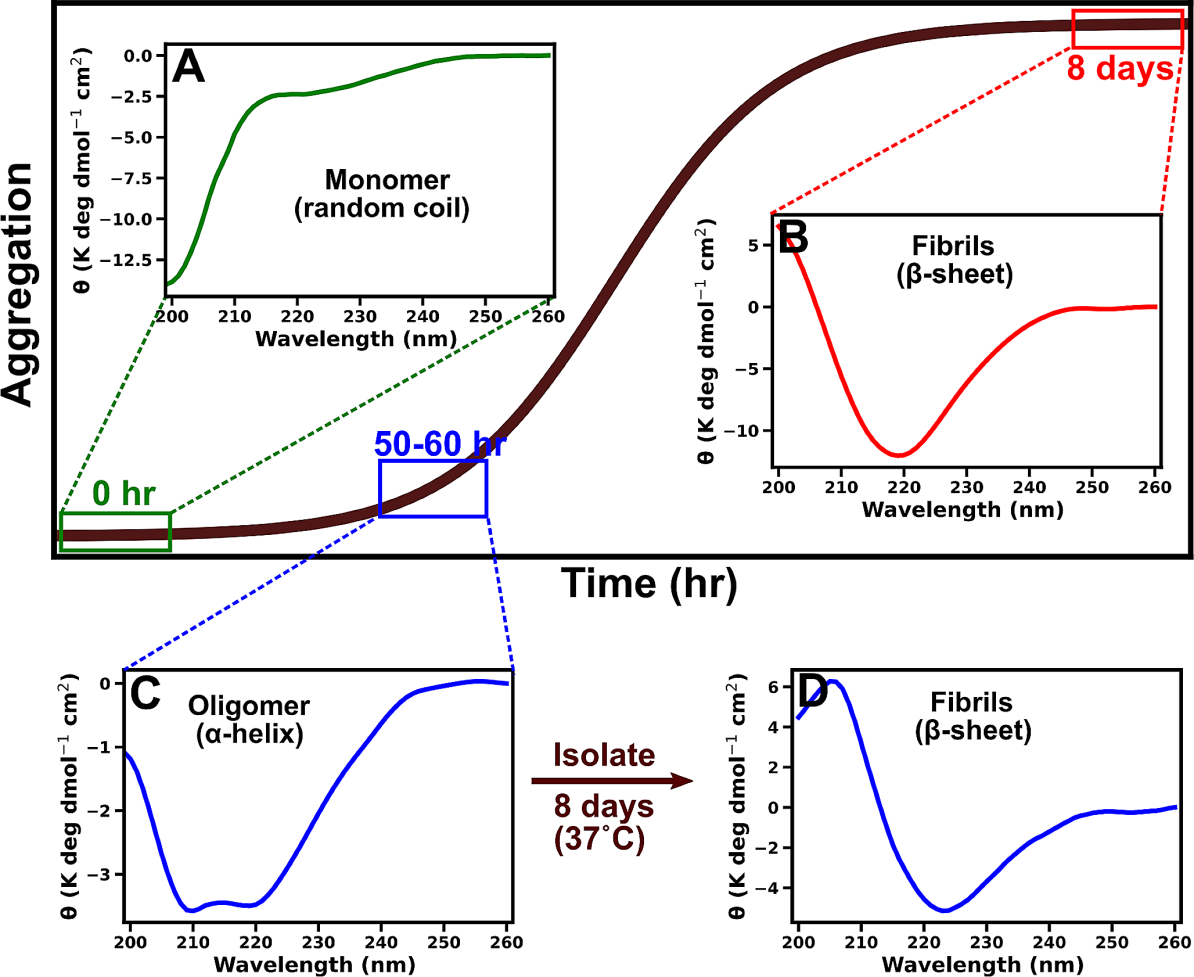
The aggregation kinetics of α-synuclein at 37˚C. At the start of fibril formation, α-synuclein are monomers (green box). These monomers are unstructured (random coil), as shown by CD spectrum (**A**). After 8 days, α-synuclein forms fibrils (red box) having classical β-sheet secondary structure evident from CD spectrum (**B**). During α-synuclein fibril formation, oligomers are observed after ~ 50–60 h of incubation (blue box). These oligomers are α-helical in CD spectrum (**C**). The oligomers were isolated and incubated for 8 days at 37˚C to mature into fibrils. The fibrils formed have β-sheet as a secondary structure (**D**)

The U-[^15^N, ^13^C] α-synuclein fibrils were obtained by incubating 300 µM of LMW in ~ 8–10 ml of 20 mM glycine-NaOH (pH 7.4) solution at 37˚C. Aggregation kinetics was continuously monitored by circular dichroism (CD) at regular intervals (Fig. 1). As soon as the CD profile migrated from the random coil to a prominent helix dip at 218 nm, the solution was centrifuged at 14,000 g for 30 min at 4˚C (Fig. 1C). The centrifugation step separates fibrillar aggregates (pellets) from oligomers and monomers (supernatant). The supernatant was collected in a fresh tube and passed through 100 kDa MWCO filters at 10,000 rpm for 30 min to separate oligomers (retentate) from monomers (flow-through). The retentate (oligomers) was collected in a fresh tube and CD showed oligomers to be α-helical (Fig. 1C). After that, the helical isolate was incubated to mature into fibrils at 37˚C for 8 days (Fig. 1D). The matured fibrils have a characteristic β-sheet secondary structure, confirmed by the CD. The resulting fibrils were ultra-centrifuged at 35,000 rpm for 1 h to obtain a pure population and labeled helix mature fibrils (HMFs). A more detailed protocol for preparing and characterizing HMF has been published in the literature (Mehra et al. 2022).

## NMR experiments

The sample was centrifuged at 2,00,000 g for 1 hour before filling into the rotor. The supernatant was removed and the pellet was filled in a 4 mm Bruker rotor using a swinging bucket centrifuge at 4000 g. A trace amount of DSS was added to the rotor before capping as an internal reference. The NMR experiments were performed on a 16.4 T (700 MHz) spectrometer with a 4 mm triple resonance (^1^H, ^13^C, ^15^N) probe at 12.5 kHz MAS frequency and sample temperature ~ 8–10˚C. All the experiments for backbone and sidechain assignments such as DARR, NCA, NCO, NCACB, NCACX, CANCO, NCOCX and CCC were recorded (Schuetz et al. 2010; Higman 2018). All the experiments were recorded on a single sample but reproducibility and stability of the sample were independently confirmed using 2D DARR and NCA spectra on three independently prepared samples. The detailed experimental parameters are reported in Table S1 of supplementary information.

## Assignments and deposition

**Fig. 2:**
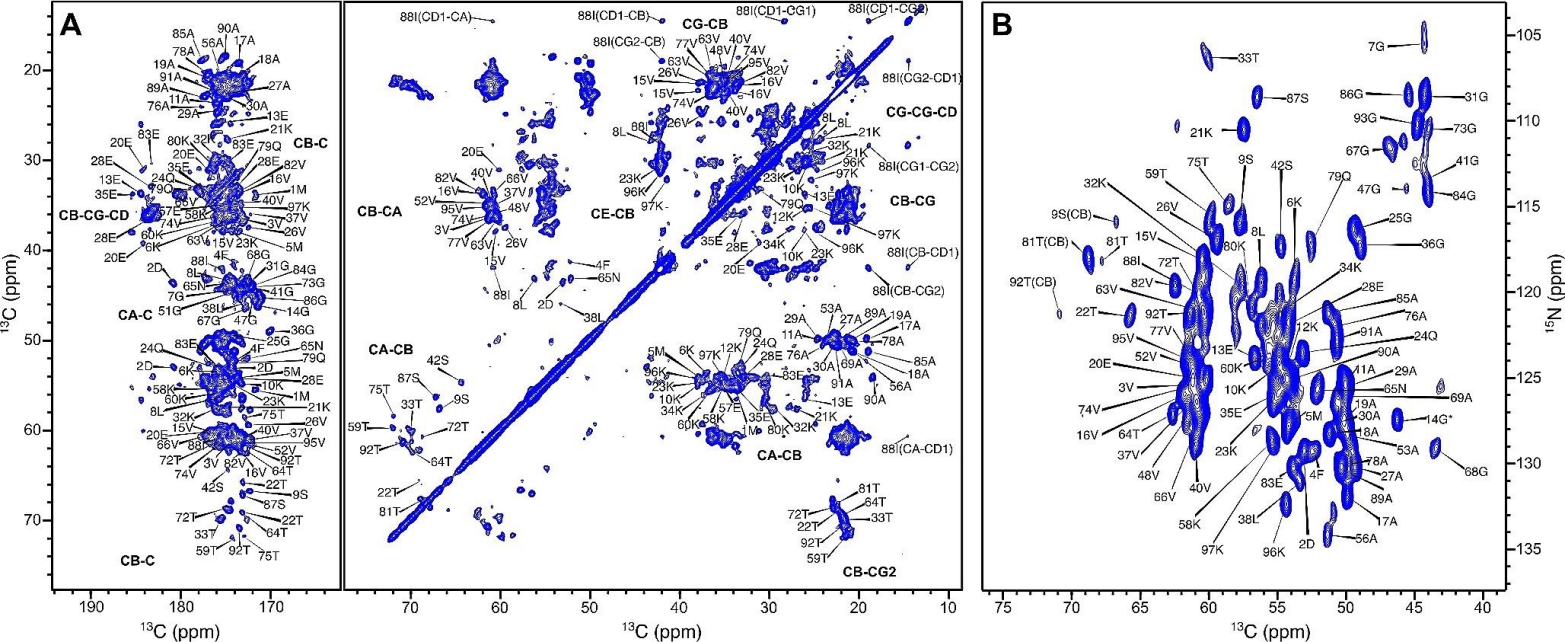
The well-resolved DARR (**A**) and NCA (**B**) spectrum. (**A**) The ^13^C-^13^C DARR spectrum of the fibril showing carbonyl and aliphatic regions. The atom types for assigned residues are mentioned in bold. The spectrum was recorded with 35 ms of mixing time on a 700 MHz spectrometer with MAS frequency at 12.5 kHz. (**B**) The NCA spectrum with assigned peaks. The 14G peak is folded and marked with an asterisk (*). A few CB peaks are visible in the spectrum and labeled as CB

**Fig. 3:**
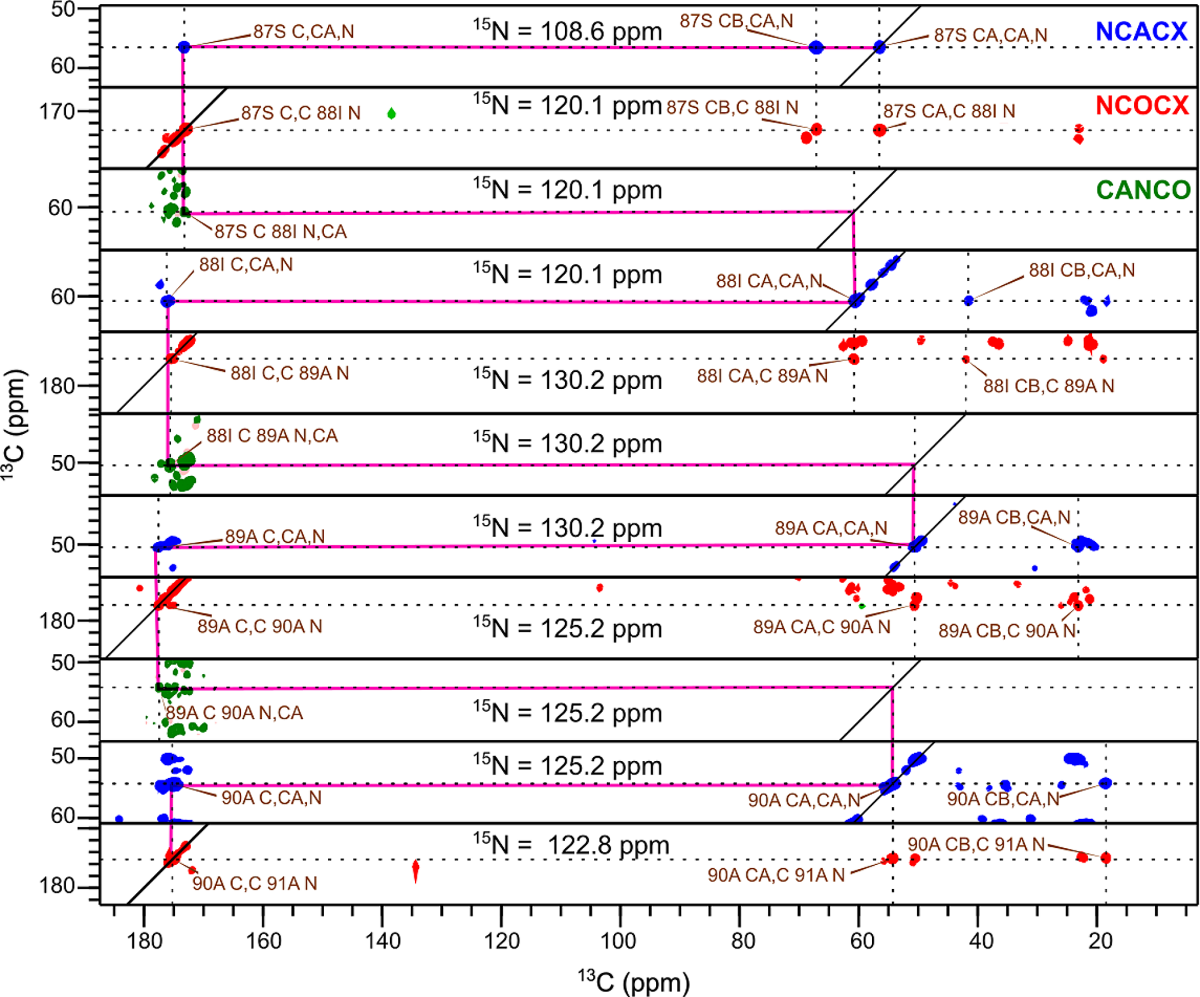
The 2D planes from 3D experiments NCACX (blue), NCOCX (red) and CANCO (green) generated in CcpNmr software (Stevens et al. 2011). The planes depict the backbone walk starting from S87. The magenta line indicates the progress of assignments

**Fig. 4:**
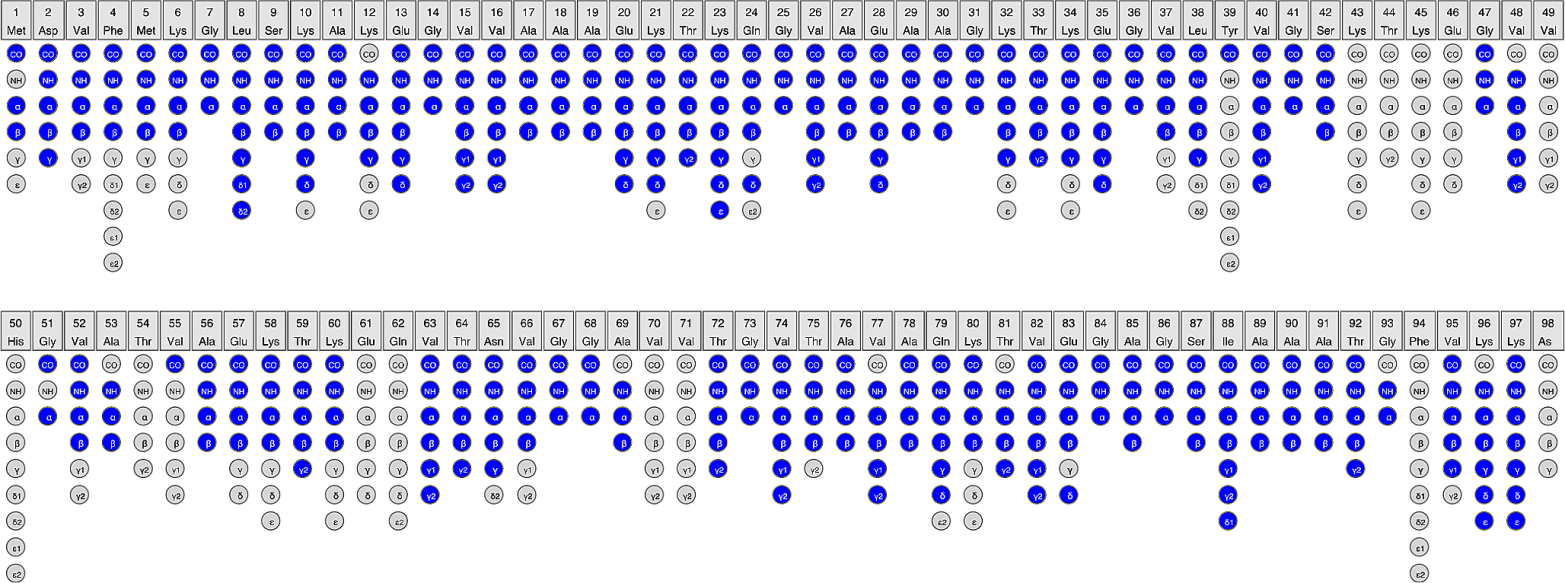
The assignment graph from CcpNmr analysis software. The assigned atoms are colored blue. The unassigned atoms are colored grey

The spectra (2D DARR and 2D NCA) acquired for HMFs are well resolved (Fig. 2). A standard set of 2D (DARR, NCA and NCO) and 3D (NCACB, NCACX, CANCO, NCOCX and CCC) spectra were acquired and used for assignment. The chemical shift assignment was performed using the standard backbone walk protocol (Schuetz et al. 2010; Higman 2018) and is shown in Fig. 3. The fibril consists of rigid and flexible regions. The C-terminal residues (98–140) are flexible and peaks of these residues are invisible in spectra employing cross-polarization (CP) for polarization transfer. The flexibility of these residues was independently confirmed by employing INEPT (Fig. S1). Out of 97 residues, 80 residues could be assigned via a backbone walk (Fig. 4) and are deposited in the BMRB under the accession number 50852. The repetitive elements in the α-synuclein sequences and low signal-to-noise for some peaks hindered the assignment. There are a few unassigned peaks in 2D NCA spectrum for threonine and glycine from which neighboring residues could not be identified. Most unassigned peaks belong to valine and lysine due to repetition and overlap. The sidechain atoms were assigned from 3D NCACX and 3D CCC spectra. Lysine and valine sidechain atoms could not be assigned unambiguously for a few residues because of a high degree of overlap.

**Fig. 5:**
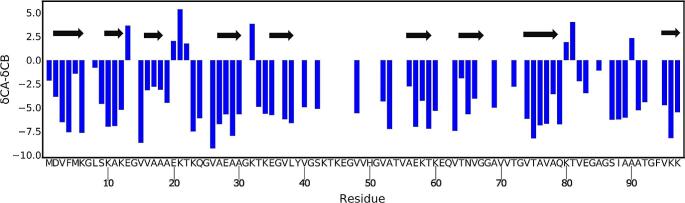
The secondary structure plot of HMFs. The blue bar indicates the CSI plot for the residues. The three sequential residues with negative values indicate the secondary structure to be β-sheet. The black arrows on the top show the secondary structure based on TALOS + prediction

The assignments were possible for residues in the N-terminal (1–40) and NAC (65–80) regions. The residues in the preNAC regions (40–60) could not be assigned with the backbone walk because of the lower signal/noise ratio. The lower signal-to-noise is most likely due to dynamics or sample heterogeneity. The chemical shifts provide information about secondary structure using the chemical shift index (CSI) (Wishart and Sykes 1994). The secondary structure of HMF was predicted by TALOS+ (Shen et al. 2009). Like all reported α-synuclein polymorphs, the secondary structure of HMF is predominantly β-sheet (Gath et al. 2011, 2014a; Comellas et al. 2011; Bousset et al. 2013; Verasdonck et al. 2016; Tuttle et al. 2016; Li et al. 2018a, b; Guerrero-Ferreira et al. 2019). The β-strands span the protein except for the preNAC region (40–60) and C-terminal (98–140) (Fig. 5). HMFs have a well-structured N-terminus with the first β-strand starting from 2nd residue (Fig. 5).

Most reported polymorphs have a rigid core spanning from residues 40–90 with flexible residues at the N-terminal and C-terminal. The absence of rigid residues in the preNAC region is reported for the ribbon polymorph (Gath et al. 2011, 2014b; Bousset et al. 2013) and polymorph amplified form ex vivo sample from DLB patient (Barclay et al. 2023; Dhavale et al. 2024) deposited in the BMRB under accession number 17498 and 51678, respectively. For most reported polymorphs (wild-type α-synuclein), residues in the N-terminal are flexible except for the ribbon polymorph. Morphologically, as the name suggests, the ribbon polymorph is ribbon-like, while HMFs are flat rod-like (Bousset et al. 2013). The ribbon and HMF polymorphs show similar chemical shifts (but not identical) and overall secondary structure. The ribbon polymorph was prepared in Tris buffer, pH 7.5, at 37˚C, while HMFs were prepared in 20 mM glycine-NaOH buffer at 37˚C. Most polymorphs prepared in vitro contain 100–150 mM of salt in the buffer (Gath et al. 2014a; Li et al. 2018b). The ribbon polymorph and HMF preparations have no salt in the buffer. The other fibril preparations without salt are in 50 mM sodium phosphate, 0.1 mM EDTA, pH 7.4, at 37˚C (Tuttle et al. 2016) and the high pH polymorph in 5 mM sodium phosphate, pH 9.0 at 37˚C (Verasdonck et al. 2016). The ex vivo fibrils extracted from MSA patients have structured residues at the N-terminal starting from G14 (PDB: 6XYO, 6XYP and 6XYQ) (Schweighauser et al. 2020). The in vitro generated polymorph 2a (PDB: 6SSX) and 2b (PDB: 6SST) have a β-strand for residues G14-Q24 in the N-terminus (Guerrero-Ferreira et al. 2019). The in vitro generated polymorph with flexible residues at the N-terminal (PDB: 7YK2), upon binding with the D1 domain of lymphocyte activation gene 3 (L3D1) results in formation of a β-strand for residues G14-G25 (PDB: 7YK8) (Zhang et al. 2023). The NAC region of all reported fibrils is rigid and consists β-strands while C-terminus is flexible for the all reported polymorphs.

The fibril prepared without intermediate isolation from the solution differs from HMFs in morphological, biophysical, biochemical and pathogenic properties. All detailed analysis for these properties is published elsewhere (Mehra et al. 2022). The chemical shift differences between two polymorphs are mapped to the preNAC region (40–60). The reported structures of α-synuclein polymorphs show that the preNAC region is involved in protofilament-protofilament interactions and most likely result in distinct properties of the two polymorphs (Li et al. 2018a; Guerrero-Ferreira et al. 2019; Schweighauser et al. 2020; Fan et al. 2023; Zhang et al. 2023). In this manuscript, we report the chemical shifts and secondary structure of a polymorph (HMF) of α-synuclein, prepared by isolating the pure α-helix oligomers population before letting them mature to fibrils. For α-synuclein, both β-sheet and random coil oligomers are also reported (Cremades et al., 2017). The α-helical oligomers, upon maturation, form the standard cross β-sheet fibrils.

## Electronic supplementary material

Below is the link to the electronic supplementary material.

Supplementary Material 1

## Data Availability

The chemical shifts are deposited in the BMRB under the accession number 50852 (https://bmrb.io/data_library/summary/index.php?bmrbId=50852).
