# Supplementary material for: Solid-state NMR assignment of α-synuclein polymorph prepared from helical intermediate
Source: Biomol NMR Assign. 2024 Jul 4;18(2):193–200. doi: 10.1007/s12104-024-10188-0 (PMC11511750; doi:10.1007/s12104-024-10188-0)
Supplement: Supplementary file 1 — Supplementary Material 1 [file 12104_2024_10188_MOESM1_ESM.docx]

**Solid-state NMR assignment of α-synuclein polymorph prepared from helical intermediate**

Sahil Ahlawat^1^, Surabhi Mehra^2^, Chandrakala M. Gowda^1^, Samir K Maji^2^, Vipin Agarwal^1*^

^1^ TIFR Centre for Interdisciplinary Sciences, Tata Institute of Fundamental Research, Hyderabad 500 046, India

^2^ Department of Biosciences and Bioengineering, Indian Institute of Technology Bombay, Powai, Mumbai-400 076, India

Supplementary Information


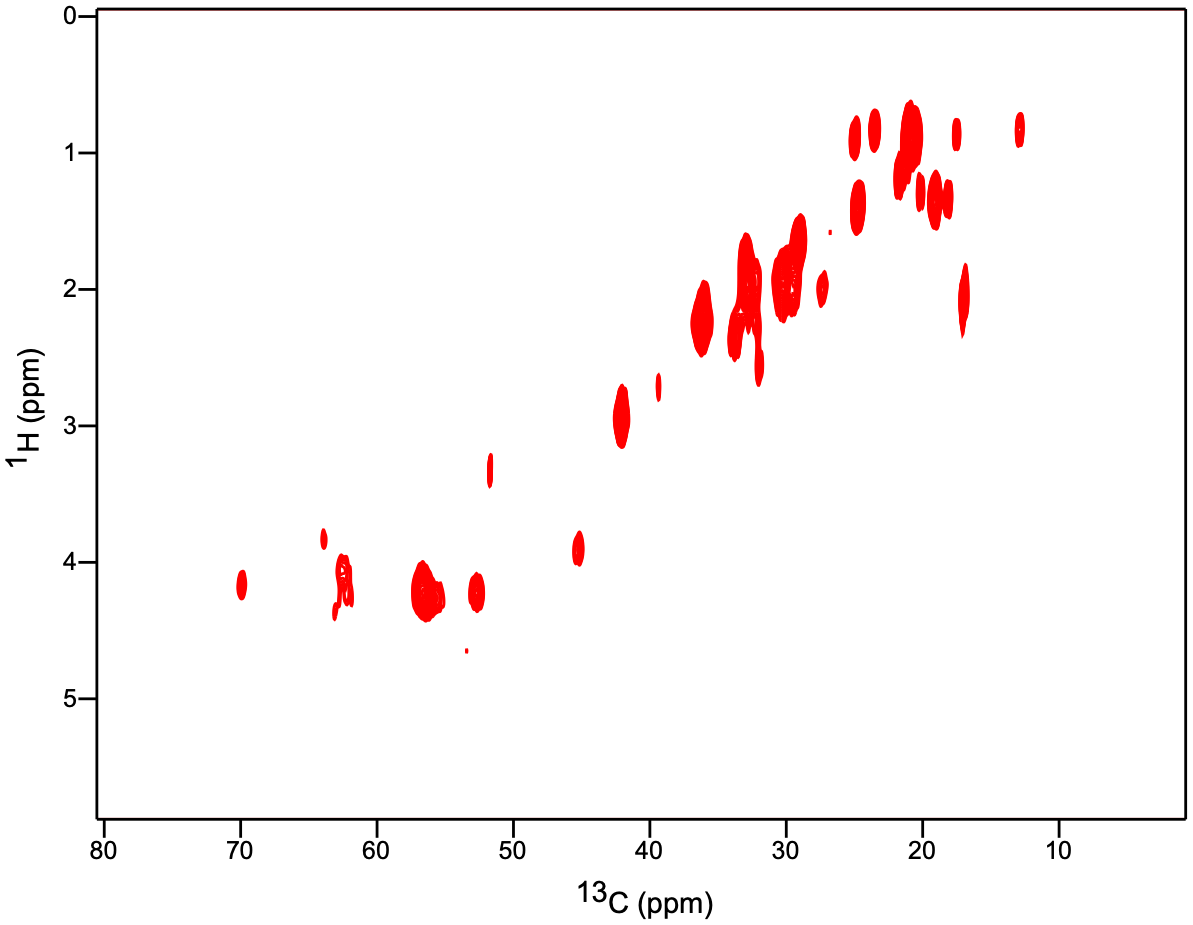


Figure 4. ^1^H-^13^C 2D reverse INEPT spectrum confirms the presence of flexible residues in the C-terminal tail

Table S1: The acquisition and processing parameters for backbone assignment of HMF polymorph on a 700 MHz spectrometer.

| **Experiment** | **DARR** | **NCA** | **3D NCACB** | **3D NCACX** | **3D NCOCX** | **3D CANCO** | **3D CCC** |
| --- | --- | --- | --- | --- | --- | --- | --- |
| ^1^H frequency | 700 MHz | 700 MHz | 700 MHz | 700 MHz | 700 MHz | 700 MHz | 700 MHz |
| Probe | 4 mm | 4 mm | 4 mm | 4 mm | 4 mm | 4 mm | 4 mm |
| MAS | 12.5 kHz | 12.5 kHz | 12.5 kHz | 12.5 kHz | 12.5 kHz | 12.5 kHz | 12.5 kHz |
| Measurement time | 19 hr | 7 hr | 45 hr | 74 hr | 89 hr | 74 hr | 155 hr |
| No. of scans | 20 | 160 | 24 | 64 | 108 | 56 | 80 |
| Recycle delay | 2.5 s | 2.5 s | 2.6 s | 2.5 s | 2.5 s | 2.5 s | 2.5 s |
|  |  |  |  |  |  |  |  |
| **Transfer 1** | **^1^H-^13^C CP** | **^1^H-^15^N CP** | **^1^H-^15^N CP** | **^1^H-^15^N CP** | **^1^H-^15^N CP** | **^1^H-^13^C CP** | **^1^H-^13^C CP** |
| RF field (kHz) | 62.93 (H)/  43.8 (C) | 56.72 (H)/  39 (N) | 56.72 (H)/  39 (N) | 56.72 (H)/  39 (N) | 56.72 (H)/  39 (N) | 64.9 (H)/  43.8 (C) | 57.86 (H)/ 44.64 (C) |
| Shape | Tangent 66-100 (H) | Tangent 66-100 (H) | Tangent 66-100 (H) | Tangent 66-100 (H) | Tangent 66-100 (H) | Tangent 66-100 (H) | Tangent 66-100 (H) |
| Carrier (ppm) | 89.2 | 113 | 114 | 113.5 | 113.5 | 49.6 | 50 |
| Time | 0.5 ms | 1.5 ms | 1.5 ms | 1.1 ms | 1.1 ms | 0.5 ms | 0.4 ms |
|  |  |  |  |  |  |  |  |
| **Transfer 2** | **DARR** | **SP-CP (N→CA)** | **SP-CP (N→CA)** | **SP-CP (N→CA)** | **SP-CP (N→CO)** | **SP-CP (CA→N)** | **DREAM (CA→CB)** |
| RF field (kHz) | 12.5 (H) | 7.71 (C)/  5.03 (N) | 7.71 (C)/  5.03 (N) | 7.71 (C)/  4.97 (N) | 5.65 (C)/  6.96 (N) | 7.42 (C)/  4.97 (N) | 5.55 (C) |
| Shape | - | Tangent 40-60 (C) | Tangent 40-60 (C) | Tangent 40-60 (C) | Tangent 40-60 (C) | Tangent 40-60 (C) | Tangent |
| Carrier (ppm) | - | 49.6 | 49.6 | 48 | 170 | 45 | 54 |
| Time | 35 ms | 5 ms | 5 ms | 5 ms | 6.5 ms | 5 ms | 2 ms |
|  |  |  |  |  |  |  |  |
| **Transfer 3** |  |  | **DREAM (CA→CB)** | **DARR** | **DARR** | **SP-CP (N→CO)** | **DARR** |
| RF field (kHz) |  |  | 5.64 (C) | 12.5 (H) | 12.5 (H) | 5.65 (C)/  6.96 (N) | 12.5 (H) |
| Shape |  |  | Tangent | - | - | Tangent 40-60 (C) |  |
| Carrier (ppm) |  |  | 37 | - | - | 170 |  |
| Time |  |  | 3 ms | 30 ms | 30 ms | 6.5 ms | 40 ms |
|  |  |  |  |  |  |  |  |
| **Decoupling** |  |  |  |  |  |  |  |
| rCW^ApA^ (kHz) | 79.3 | 79.3 | 79.3 | 79.3 | 79.3 | 80.6 | 75.5 |
| CW (kHz) |  | 79.3 | 79.3 | 79.3 | 79.3 | 80.6 | 75.7 |
|  |  |  |  |  |  |  |  |
| **t_1_ evolution** | **^13^C** | **^15^N** | **^15^N** | **^15^N** | **^15^N** | **^13^CA** | **^13^CA** |
| Points | 1250 | 64 | 40 | 34 | 36 | 56 | 34 |
| SW (ppm) | 250 | 32 | 32 | 33 | 33 | 25 | 24 |
| Carrier (ppm) | 89.2 | 113 | 114 | 113.5 | 113.5 | 49.6 | 50 |
| Acquisition time (ms) | 14.2 | 14 | 10.2 | 7.2 | 7.6 | 6.3 | 4.0 |
| td processing | 4096 | 512 | 256 | 256 | 256 | 256 | 128 |
| Window function | qsine 3 | qsine 3 | qsine 3 | qsine 3 | qsine 3 | qsine 3 | qsine 2 |
|  |  |  |  |  |  |  |  |
| **t_2_ evolution** | **^13^C** | **^13^CA** | **^13^CA** | **^13^CA** | **^13^CO** | **^15^N** | **^13^C** |
| Points | 1776 | 1778 | 64 | 50 | 32 | 32 | 80 |
| SW (ppm) | 338 | 338 | 338 | 25 | 12 | 32 | 56 |
| Carrier (ppm) | 89.2 | 49.6 | 49.6 | 46 | 164 | 113 | 40 |
| Acquisition time (ms) | 14.9 | 14.9 | 7.8 | 5.7 | 7.5 | 7.0 | 4.0 |
| td processing | 8192 | 4096 | 256 | 256 | 256 | 256 | 256 |
| Window function | qsine 3 | qsine 3 | qsine 3 | qsine 3 | qsine 3 | qsine 3 | qsine 2 |
|  |  |  |  |  |  |  |  |
| **t_3_ evolution** |  |  | **^13^C** | **^13^C** | **^13^C** | **^13^CO** | **^13^C** |
| Points |  |  | 1422 | 1422 | 1422 | 1422 | 1422 |
| SW (ppm) |  |  | 338 | 338 | 338 | 338 | 338 |
| Carrier (ppm) |  |  | 49.6 | 46.5 | 46.5 | 49.6 | 50 |
| Acquisition time (ms) |  |  | 11.9 | 11.9 | 11.9 | 11.9 | 11.9 |
| td processing |  |  | 8192 | 4096 | 4096 | 4096 | 4096 |
| Window function |  |  | qsine 2 | qsine 2 | qsine 2 | qsine 2.5 | qsine 2 |
